# Personal Appearance and the Culture of Beauty

**Published:** 1877-11-20

**Authors:** 


Personal Appearance and the Culture of Beauty, with hints as to Character, by T. S. Sozinskey, M. D., Ph. D., Philadelphia. Allen, Lane & Scott, 233 South Fifth Street.

This a neat and pretty little volume of 187 pages, on a subject of considerable interest to the general as well as the professional reader.

